# Sustainable MXene Synthesis via Molten Salt Method and Nano-Silicon Coating for Enhanced Lithium-Ion Battery Performance

**DOI:** 10.3390/molecules30040812

**Published:** 2025-02-10

**Authors:** Hansu Kim, Yunki Jung, Wonhwa Lee, Young-Pyo Jeon, Jin-Yong Hong, Jea Uk Lee

**Affiliations:** 1 Department of Advanced Materials Engineering for Information and Electronics, Integrated Education Institute for Frontier Science and Technology (BK21 Four), Kyung Hee University, 1732 Deogyeong-Street, Giheung-gu, Yongin-si 17104, Republic of Korea; hansu@khu.ac.kr (H.K.); yunki0930@khu.ac.kr (Y.J.); lwh980304@khu.ac.kr (W.L.); 2 Hydrogen & C1 Gas Research Center, Korea Research Institute of Chemical Technology (KRICT), 141 Gajeong-ro, Yuseong-gu, Daejeon-si 34114, Republic of Korea; ypjeon@krict.re.kr; 3 Advanced Materials and Chemical Engineering, University of Science and Technology (UST), 217, Gajeong-ro, Yuseong-gu, Daejeon-si 34113, Republic of Korea

**Keywords:** MXene, eco-friendly, molten salt, nano-silicon, lithium-ion batteries

## Abstract

MXenes, a family of 2D transition metal carbides, nitrides, and carbonitrides, have attracted significant attention due to their exceptional physicochemical properties and electrochemical performance, making them highly promising for diverse applications, particularly in energy storage. Despite notable advancements, MXene synthesis remains a critical challenge, as conventional methods often rely on hazardous hydrofluoric acid-based processes, posing substantial environmental and safety risks. In this study, we present an eco-friendly synthesis approach for MXenes using molten salt processes, which offer a safer, sustainable alternative while enabling scalable production. Additionally, we explore the development of high-performance battery anodes by fabricating nanocomposites of nano-silicon and MXene, followed by a bio-inspired polydopamine coating and carbonization process. This innovative strategy not only enhances the structural stability and electrochemical performance of the anodes but also aligns with environmentally conscious design principles. Our findings demonstrate the potential of eco-friendly MXene synthesis and nanocomposite materials in advancing sustainable energy storage technologies.

## 1. Introduction

**Figure 1:**
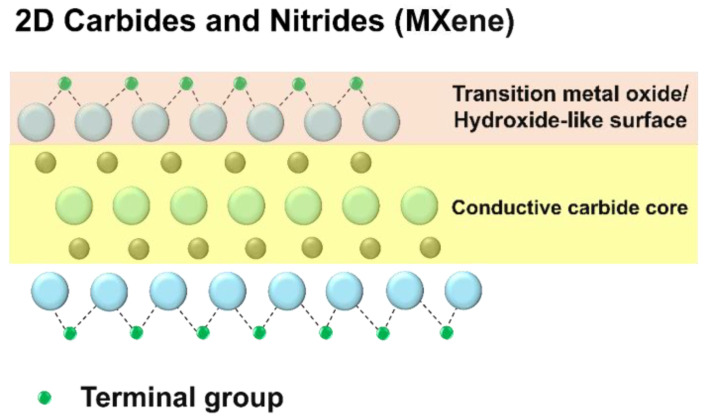
Structure of 2D carbide and nitride (MXene).

The rapid transition towards sustainable energy solutions has significantly increased the demand for innovative materials capable of enabling high-efficiency and environmentally friendly energy storage systems. In this context, titanium carbide MXenes, a class of two-dimensional materials, have gained considerable attention due to their exceptional electrical conductivity, hydrophilicity, and versatile surface functionalities. These properties make MXenes ideal candidates for applications in energy storage devices such as lithium-ion batteries (LIBs) and supercapacitors [1,2,3]. Derived from MAX phases with the general formula MAX, MXenes are synthesized by selectively etching the A layer, resulting in their unique two-dimensional structure [4,5,6,7], as shown in Figure 1.

MXenes possess tunable electrical conductivity and mechanical strength, which make them well-suited for diverse applications, including energy storage, sensors, and electromagnetic shielding [8,9,10,11,12,13,14]. However, conventional HF-based synthesis methods introduce fluorine terminal groups that adversely affect the performance of MXenes in energy storage applications [15,16,17,18,19,20,21]. To address these challenges, alternative synthesis methods such as molten salt etching have been developed. These methods provide safer and more environmentally friendly approaches while enabling adjustable surface properties and reducing environmental impact [13,22,23,24,25,26].

Simultaneously, silicon-based anodes have emerged as promising next-generation materials for LIBs due to their significantly higher theoretical capacity of 4200 mAh/g, far surpassing that of graphite [27,28,29,30]. Despite their advantages, silicon anodes face substantial challenges, including mechanical instability and performance degradation caused by volume expansion during charge–discharge cycles [31,32,33,34,35]. Proposed strategies to mitigate these issues include the use of nano-silicon and silicon suboxide (SiOx); however, these approaches are limited by low conductivity and particle aggregation [36,37,38,39].

Polydopamine (PDA)-derived carbon coating has been introduced as a promising solution to enhance the stability of silicon anodes. PDA forms a flexible and conductive carbon layer that accommodates the volume changes of silicon while improving its electrical conductivity [40,41,42]. The in situ coating process is cost-effective, environmentally friendly, and suitable for large-scale commercialization [43,44,45]. Recent studies have further optimized PDA coatings by adjusting thickness and carbonization levels, significantly improving the performance and cycle life of silicon anodes [46,47,48,49].

By integrating MXenes synthesized via molten salt methods with PDA-coated silicon anodes, this study aimed to advance the development of eco-friendly and high-performance energy storage materials. These innovations address critical challenges in energy density and device stability, paving the way for their broader application in sustainable energy technologies.

## 2. Materials and Methods

### 2.1. Materials

Titanium carbide MXene (${Ti}_{3}C_{2})$ was synthesized using the MAX phase ${Ti}_{3}{AlC}_{2}$ (99.999% purity, 325 mesh, powder, Uninanotech, Yongin-si, Republic of Korea) along with ${ZnCl}_{2}$ (99.0% purity, powder, Samchun chemicals, Pohang-si, Republic of Korea). ${ZnCl}_{2}$ has a low melting point (290 °C) and high solubility in water (432 g/100 g). Next, lithium chloride (LiCl, 99% purity, powder, Sigma-Aldrich, Saint Louis, MO, USA) and *N*-methylformamide (NMF, 99.9% purity, Sigma-Aldrich) were used for intercalation for efficient delamination. For the Si-MXene composite, 2 g of Silicon Nano Particles (SiNP, 50 nm, powder, Avention, Concord, MA, USA) was used. Dopamine hydrochloride (DA, 98% purity, powder, Sigma-Aldrich) was used for carbon coating on the Si-MXene composite [50].

### 2.2. MAX Etching for Multi-Layer MXene

**Figure 2:**
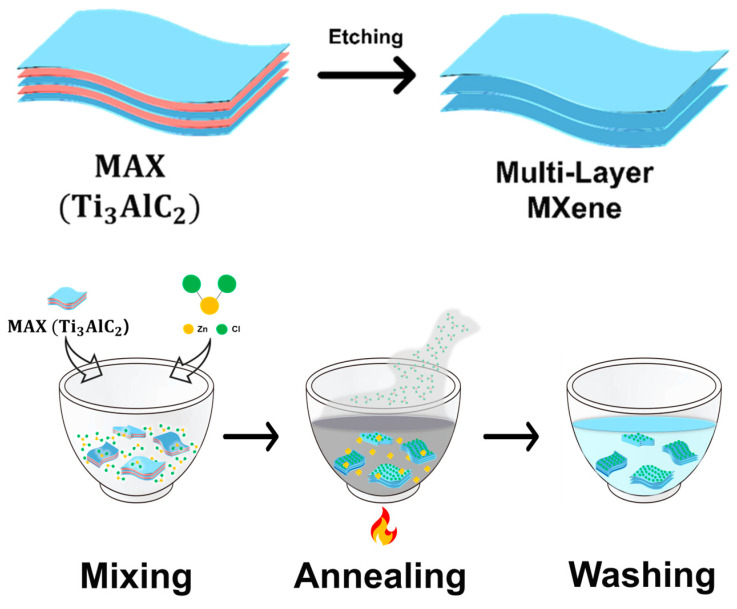
Schematic of Etching for multi-layer MXene synthesis processes.

Multi-layer MXene was synthesized by mixing the MAX phase ${Ti}_{3}{AlC}_{2}$ and ${ZnCl}_{2}$ at a ratio of 1:6 (Figure 2). The starting material was mixed thoroughly using a mortar under the protection of Argon in a glove box. The mixtures were annealed at 700 °C in Argon for 4 h. Thus, the Lewis acid ${Zn}^{2+}$ oxidized Al to form multi-layer MXenes (${Ti}_{3}C_{2}Cl)$. Then, the solution was washed with 0.1 M HCl solution (Samchun chemicals) at 300 rpm for 5 h to remove unreacted ${ZnCl}_{2}$ and Zn. The acid-washed MXene was transferred into a centrifuge tube filled with deionized water and was washed with a centrifuge (1248, Labogene, Lillerød, Denmark) at 1400 rcf for 5 min three times. After the centrifuge wash, the final product was collected using a polyvinylidene fluoride membrane (0.2 μm, 47 mm) and dried under vacuum at 40 °C.

### 2.3. Delamination for Single-Layer MXene

**Figure 3:**
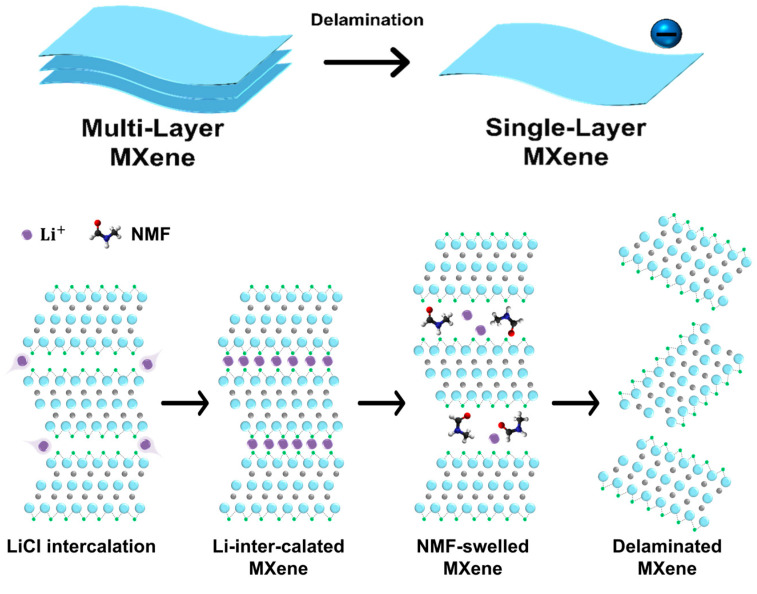
Schematic of single-layer MXene synthesis processes.

Multi-layer MXene produced using the Lewis acid method can be delaminated using lithium salts in nonaqueous solvents, such as dimethyl sulfoxide (DMSO) (Figure 3). First, 1.2 g of LiCl was added to 10 mL of DMSO (99.9% purity, Sigma-Aldrich) in 30 mL glass vials and dissolved by stirring with a PTFE-coated magnetic stir bar for 10 min. Then, 3 g of multi-layer MXene was added to the solution and stirred at 1000 rpm for 24 h for intercalation. After the treatment with LiCl/DMSO, the ${Li}^{+}$-intercalated multi-layer MXene was transferred into a 50 mL centrifuge tube and centrifuged at 1500 RCF for 5 min to remove DMSO solvent and excess lithium chloride salt. After removing excess ${Li}^{+}$, the sediment was redispersed into 15 mL of *N*-methylformamide (NMF, 99.9% purity, Sigma-Aldrich) for delamination. NMF with a high dielectric constant effectively screens the electrostatic interactions between negatively charged MXene layers, intercalates Li cations, and facilitates better solvation of lithium cations [51]. This was evidenced in this study by the dark supernatant that was observed following centrifugation at 250 RCF for 5 min. This supernatant contained single-layer MXene with large flake sizes, which was collected for further analysis. To increase the yield of delamination, bath sonication (JAC Ultrasonic, KODO, Seoul, Republic of Korea) was performed at room temperature for 20 min. Subsequently, centrifugation was performed at 11,200 RCF for 10 min to facilitate solvent exchange with isopropyl alcohol (IPA) or deionized water.

### 2.4. Self-Assembly of Si-MXene Composite and Polydopamine Coating

To prepare modified silicon nanoparticles, 2 g of silicon nanoparticles (SiNP, 50 nm, Avention) were dispersed in 500 mL of deionized water and stirred continuously. Subsequently, 15 mL of a 20 wt% aqueous solution of hexadecyltrimethylammonium bromide (CTAB, 98% purity, Sigma-Aldrich) was gradually introduced into the SiNP dispersion while stirring for 1 h, followed by an additional 1 h of ultrasonication. The resulting suspension was subjected to centrifugation at 7000 rpm for 5 min and washed repeatedly with deionized water over five cycles. The CTAB-modified SiNPs were designated as CTAB@Si.

**Figure 4:**
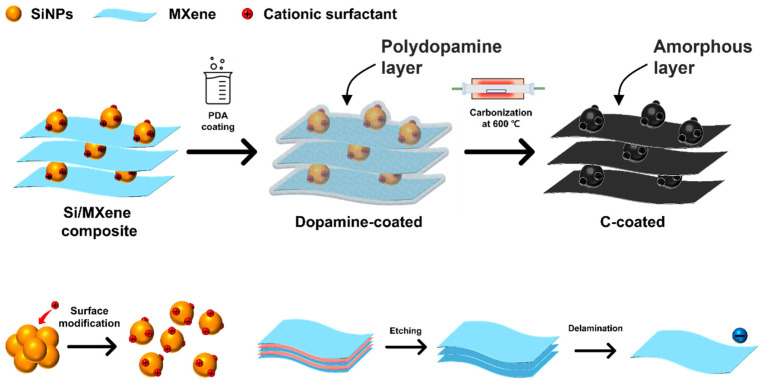
Schematic of formation of Si-MXene composite and polydopamine coating.

For the preparation of the composite material, 2 g of the CTAB@Si and 400 mg of single-layer MXene were dispersed in 400 mL of Tris buffer (0.01 M, pH 8.7). This mixture was stirred at 500 rpm for 6 h to achieve a homogeneous suspension. Subsequently, 2 g of dopamine hydrochloride (DA, Sigma-Aldrich) was added, and the reaction was allowed to proceed under continuous stirring at 25 °C for 24 h. The modification of SiNPs with CTAB effectively minimized agglomeration, enabling uniform integration with single-layer MXene through electrostatic self-assembly. After coating the Si-MXene composite with PDA, the dried material was subjected to sintering in an argon atmosphere at 600 °C for 5 h to produce the final Si-MXene/PDA composite (Figure 4).

As a control sample, Si/PDA was also prepared according to the following process: 2 g of SiNPs was dispersed in 400 mL of Tris buffer (0.01 M, pH 8.7). This mixture was stirred at 500 rpm for 6 h to achieve a homogeneous suspension. Subsequently, 2 g of DA was added, and the reaction was allowed to proceed under continuous stirring at 25 °C for 24 h. After coating the SiNPs with PDA, the dried material was subjected to sintering in an argon atmosphere at 600 °C for 5 h to produce the final Si/PDA.

### 2.5. Characterizations

The phase composition and crystal structures of the samples were characterized using an X-ray diffractometer (D8 Advance, Bruker, Berlin, Germany) with a scanning rate of 6°/min. Raman spectra were obtained using a Raman microscope (inVia Raman microscope, Renishaw, Kingswood, UK). Zeta potential measurements were performed with a laser particle size analyzer (M 3000, Marvin, Herresbach, Germany). The surface chemistry and elemental states were analyzed through X-ray photoelectron spectroscopy (XPS, K-Alpha, Thermo Fisher Scientific, Waltham, MA, USA). Microstructural analysis was carried out using a scanning electron microscope (SEM, Merlin, Zeiss, Oberkochen, Germany) equipped with an energy-dispersive spectroscopy (EDS) system. High-resolution imaging and compositional mapping were conducted using a Cs-corrected transmission electron microscope (Cs-TEM, Jeol, Tokyo, Japan) with an integrated SuperX EDS detector. Surface topography was studied with atomic force microscopy (AFM, GmbH, Westfalen, Germany), while powder resistivity was measured using a resistivity analyzer (HPRM-FA2, HanTech, Ulsan, Republic of Korea). The electrochemical impedance spectrum (EIS) was obtained using an Electrochemical Interface and Impedance Analyzer (CompactStat.h, Ivium Technologies, Eindhoven, The Netherlands).

### 2.6. Fabrication of Si-MXene Composite Andoe for Half Cell

A slurry was prepared by mixing active materials, acetylene black, and sodium alginate at a mass ratio of 8:1:1. The mixture was applied evenly onto a copper foil substrate and dried at 80 °C for 5 h. Circular electrodes with a diameter of 14.0 mm were then punched out from the coated foil, with an active material loading of approximately 1.1–1.5 mg/cm^2^. Electrode composite density (*ρ*) was calculated using the following formula:

$$\rho =\frac{electrodeweight(g)}{electrodearea\left({cm}^{2}\right)\times electrodethickness(cm)}$$

To achieve uniform densities across all electrodes, the thickness was adjusted based on the above calculation methods.

Lithium metal served as the counter electrode, while MS PCS513 (Enerever Battery Solution, Wanju, Republic of Korea) was utilized as the separator. The electrolyte consisted of a solution of 1.0 M LiPF6 dissolved a mixture comprising a 1:1:1 volumetric ratio of ethylene carbonate (EC), diethyl carbonate (DEC), and fluoroethylene carbonate (FEC). All assembly steps were conducted within an argon-filled glove box, using Neba 2032 coin cell cases. Electrochemical charge and discharge profiles were recorded within a voltage range of 0.01–2.0 V (vs. Li/Li^+^) at room temperature. The electrochemical performance of the coin cells was assessed using a multichannel battery tester (WBCS3000, WonATech, Seoul, Republic of Korea).

## 3. Results and Discussion

### 3.1. Ecofriendly Synthesis of MXene

#### 3.1.1. Synthesis of Multi-Layer MXene

**Figure 5:**
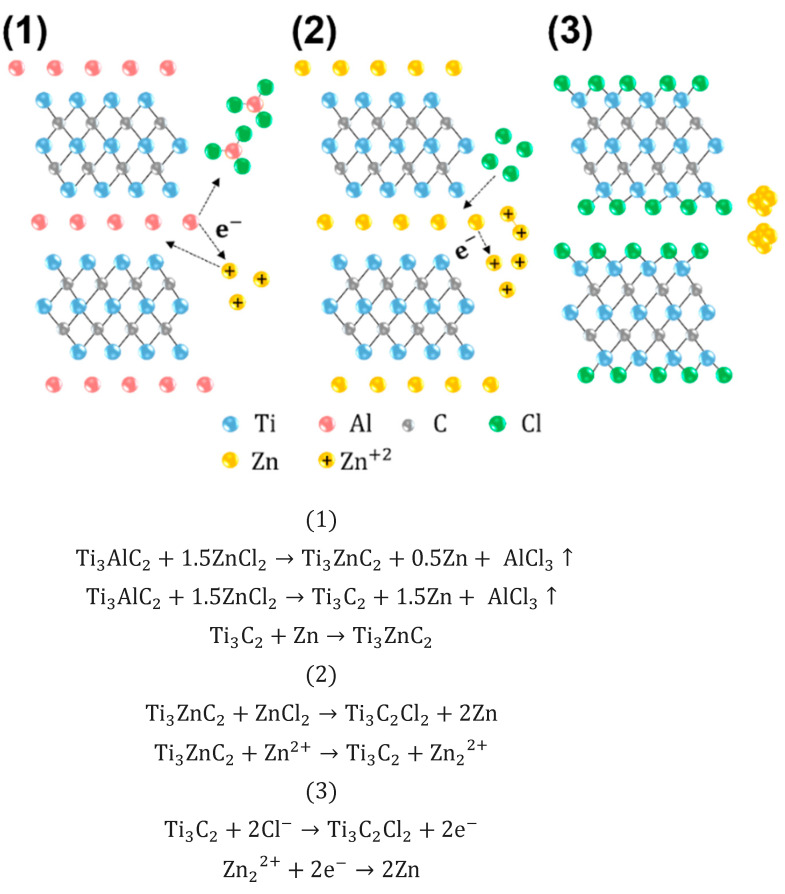
Schematic and Mechanism of etching process (1–3) for multi-layer MXene.

Multi-layer $MXene\;({Ti}_{3}C_{2}{Cl}_{2})$ was synthesized by selective etching of the aluminum layers from the MAX $({Ti}_{3}AlC_{2}$) phase precursor using a molten salt etchant with the reactions that follow, as shown in Figure 5. Reaction 1 is a general reaction, well known for the properties of ZnCl_2_, which has a melting point of approximately 280 °C and exists as Zn^2+^ ions and the ${{ZnCl}_{4}}^{2-}$ tetrahedron in its molten state. The coordinately unsaturated ${Zn}^{2+}$ ions act as strong acceptors of ${Cl}^{-}$ and electrons, functioning as Lewis acids in the $Zn{Cl}_{2}$ molten salt. In this acidic environment, the weakly bonded Al atoms in ${Ti}_{3}AlC_{2}$ can be easily converted to ${Al}^{3+}$ by a redox reaction. This leads to the formation of the Ti_3_ZnC_2_ phase, where the exchange between Al and Zn occurs. If the amount of ZnCl_2_ continues to increase after the reaction, the Zn in the Zn-MAX phase will eventually be released in metallic form. The formation of this Zn-MAX phase suggests that the exchange mechanism could also be applicable to unexplored MAX phases, involving traditional Al-MAX phases and late-transition-metal halides [52]. The as-produced ${Al}^{3+}$ would further bond with ${Cl}^{-}\;$to form $Al{Cl}_{3}$, which has a boiling point of ~180 °C and is expected to rapidly evaporate at the reaction temperature. As reactions 2 and 3 occur in the absence of oxygen or water, the resulting multi-layer MXene exhibits a uniform Cl termination. Cl terminations introduced via molten salt synthesis significantly improve the electrical conductivity, ion transport kinetics, and electrochemical stability of MXenes. Unlike HF-based synthesis, which results in F terminations that hinder electron mobility due to their high electronegativity, Cl terminations preserve the metallic properties and enhance electron transport. Additionally, Cl-terminated MXenes exhibit reduced oxygen content, which mitigates the formation of insulating regions and further enhances conductivity. The larger atomic radius of Cl also increases interlayer spacing, facilitating ion intercalation and improving overall electrochemical performance. Moreover, Cl terminations stabilize surface chemistry, reduce side reactions during lithium-ion intercalation, and optimize surface reactivity, leading to better electrochemical processes. In the final step, residual Zn metal present in the as-synthesized multi-layer MXene was completely removed by thorough washing with 0.1 M HCl, as shown in reaction 4.

**Figure 6:**
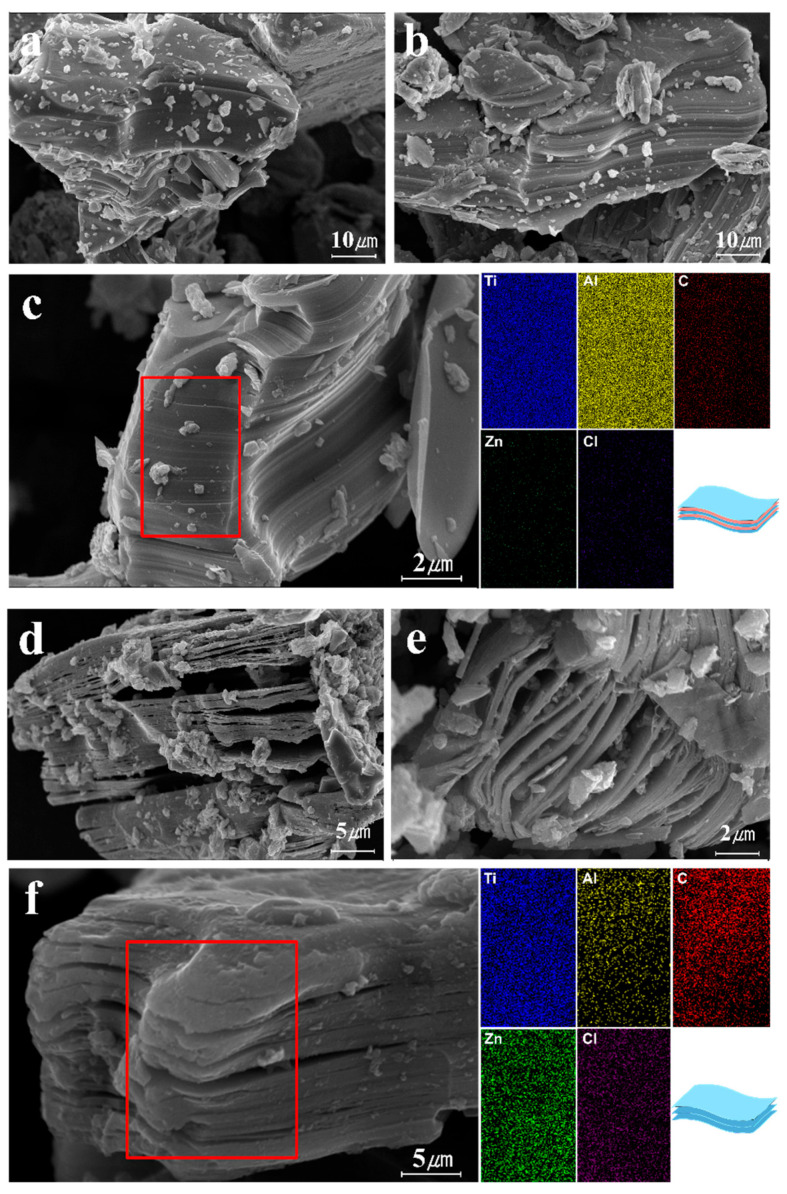
FE-SEM images and EDS mapping of (**a**–**c**) MAX and (**d**–**f**) multi-layer MXene.

FE-SEM images of powder morphology displayed by MAX and multi-layer MXene are shown in Figure 6. Figure 6a–c show typical images of the MAX phase. In the MAX phase, the layers of M (Ti), A (Al), and X (C) were observed to be stacked in multiple layers (${Ti}_{3}AlC_{2})$. However, the A (Al) layer was removed after molten salt etching, resulting in an accordion-like structure, as shown in Figure 6d–f. It was observed that the remaining Ti layers and C layers of MXenes were connected by van der Waals forces [53]. Subsequent EDS mapping confirmed that the Al layers disappeared and that Zn and Cl were formed after etching with the molten salt etchant ($Zn{Cl}_{2})$.

#### 3.1.2. Synthesis of Single-Layer MXene

**Figure 7:**
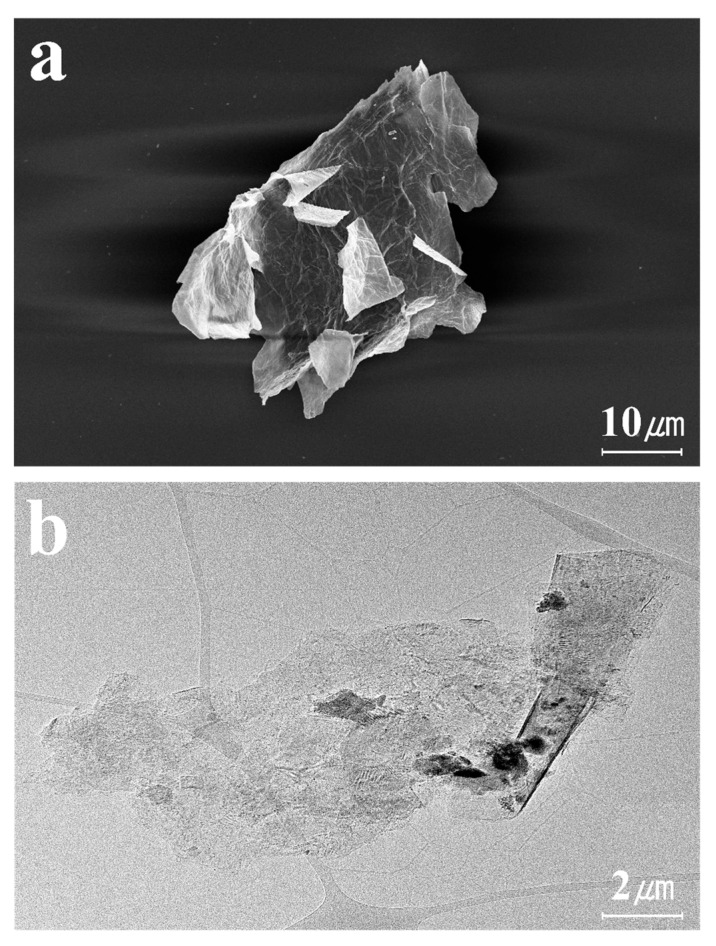
(**a**) FE-SEM and (**b**) Cs-TEM images of single-layer MXene.

**Figure 8:**
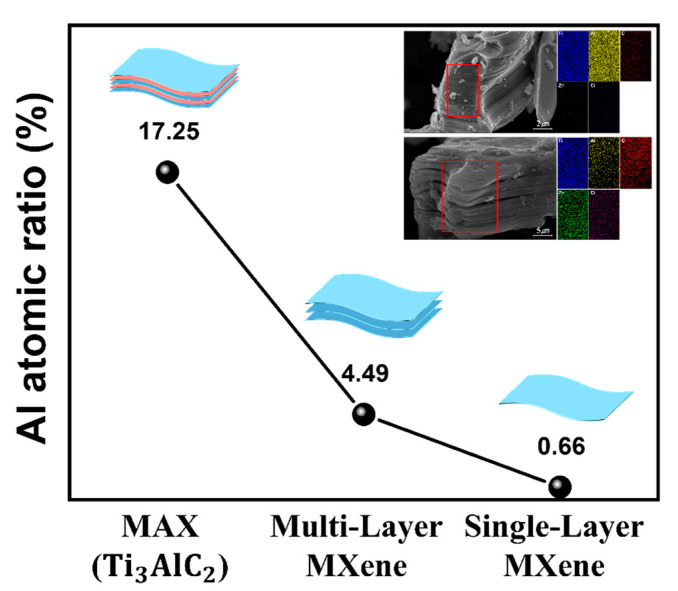
Al atomic ratio graph of MAX, multi-layer MXene, and single-layer MXene.

**Figure 9:**
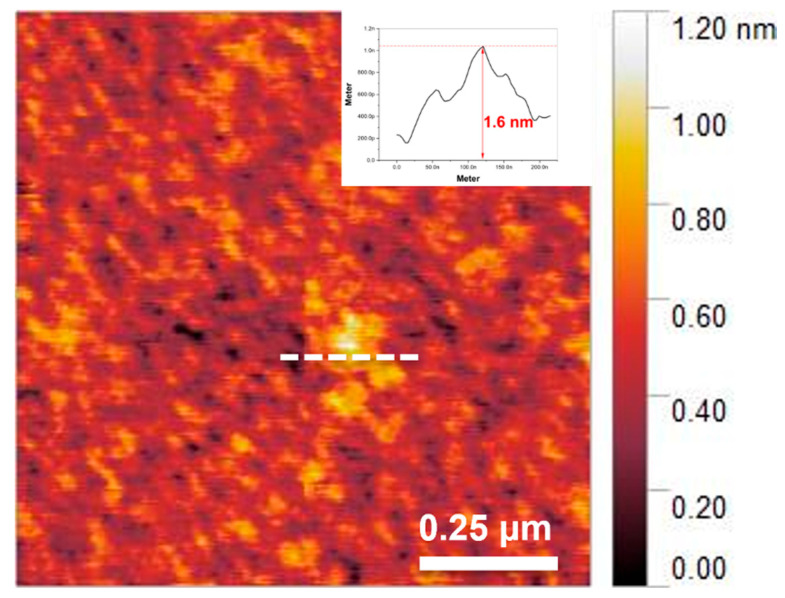
AFM image of single-layer MXene.

After the delamination process, the single-layer MXene produced, divided into individual layers, can be observed in Figure 7a,b. The delaminated single-layer MXene exhibits an extremely thin, sheet-like morphology and offers a higher specific surface area compared to MAX phases and multi-layer MXenes. EDS mapping was conducted to characterize the elemental composition of the synthesized MXene. As the etching and delamination processes were performed sequentially, the Al content decreased from 17.25% to 4.49%, and finally to 0.66%, as shown in Figure 8. The residual Al after etching may be attributed to the formation of Al byproducts, which are challenging to remove completely [54]. This indicates that the Al layer was almost completely removed after the final delamination process. Subsequently, the AFM image confirmed that the final MXene had a thickness of approximately 1.6 nm, as shown in Figure 9. Given that the thickness of a single-layer MXene is typically 1–2 nm, this measurement corresponds to a single-layer MXene.

#### 3.1.3. Analysis of Ecofriendly Synthesized MXenes

**Figure 10:**
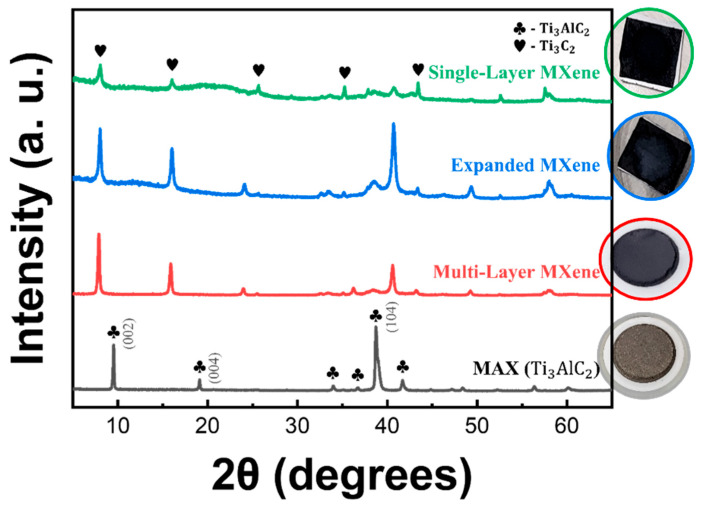
X-ray diffraction patterns of MAX (gray), multi-layer MXene (red), expanded MXene (blue), and single-layer MXene (green).

X-ray diffraction patterns of all samples from the MAX phase to the single-layer MXene are shown in Figure 10. In the MAX phase, multiple titanium aluminum carbide reflections, including (002), (004), and (104) planes, were identified. Following the etching process, the successful removal of the Al layer was validated by a reduction exceeding 50% in the intensity of the (104) reflection in the multi-layer MXene structure. This transformation was further corroborated by a visible color change from gray in the MAX phase powder to charcoal black in the etched powder. Additionally, the leftward shift of the (002) and (004) reflections indicates an expansion in the interlayer spacing of the MXene due to the removal of the Al layer. The d-spacing value based on the (002) reflection increases from 9.28 Å in the MAX phase to 11.08 Å in the multi-layer MXene, while for the (004) reflection, it decreases from 5.69 Å in the MAX phase to 4.64 Å in the multi-layer MXene. The increase in d-spacing observed in the (002) diffraction pattern is twice as large as that observed in the (004) pattern. Furthermore, the (004) peak displays a larger shift compared to the (002) peak, aligning well with theoretical predictions [55]. Lithium-ion intercalation was subsequently employed to facilitate delamination. The expanded MXene diffraction pattern showed an increase in the intensity of the (104) reflection, indicating successful structural expansion. In the case of the single-layer MXene, the overall reduction in peak intensity suggests the formation of monolayer structures. Additionally, the significant attenuation of the (104) reflection confirmed the effective removal of the Al layer as well as the intercalated lithium ions. Multiple titanium carbide phases were observed in the diffraction data, providing structural evidence for the formation of single layers. Visually, the charcoal-black multi-layer MXene transformed into a black single-layer MXene following the delamination process.

**Figure 11:**
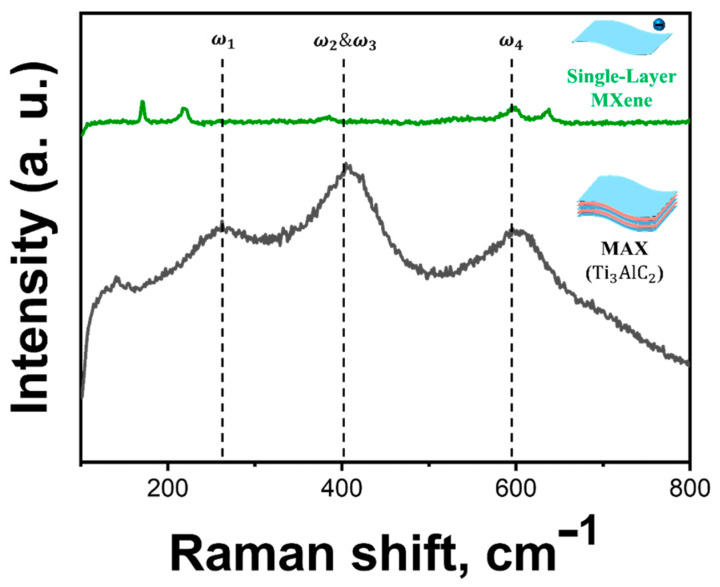
Raman spectra of MAX (gray) and single-layer MXene (green).

Figure 11 shows the Raman spectra of the MAX phase and the single-layer MXene. The 100–800 cm^−1^ region corresponds to the lattice vibrations (phonons). Generally, the Raman spectra of MAX and MXene exhibit only slight differences [56,57]. The peaks labelled as ω_1_, ω_2_&ω_3_, and ω_4_ in the spectrum were located around 269, 407, and 590 cm^−1^, respectively. These peaks are key features of MAX and matched well with those reported in the literature, attributed to shear and longitudinal oscillations of Ti and the Al atoms [58,59,60]. Specifically, ω_1_ is associated with vibrations of Al; its disappearance in the of MXene spectrum correlates with the substantial etching of Al atoms, resulting in the formation of a MXene structure [61]. By analyzing the Raman spectra around the 200 cm^−1^ region, the influence of surface terminations can be evaluated. Fluorine-terminated MXenes synthesized through conventional HF etching methods exhibit a strong Raman band at approximately 200 cm^−1^, attributed to F-Ti vibrations. This study focused on fluorine-free MXenes synthesized using the molten salt etching method, where no peak was observed at 200 cm^−1^, confirming the absence of fluorine-related surface terminations. These results are supported by various reports in the literature, and can be taken to signify the successful ecofriendly synthesis of single-layer MXene [62].

**Figure 12:**
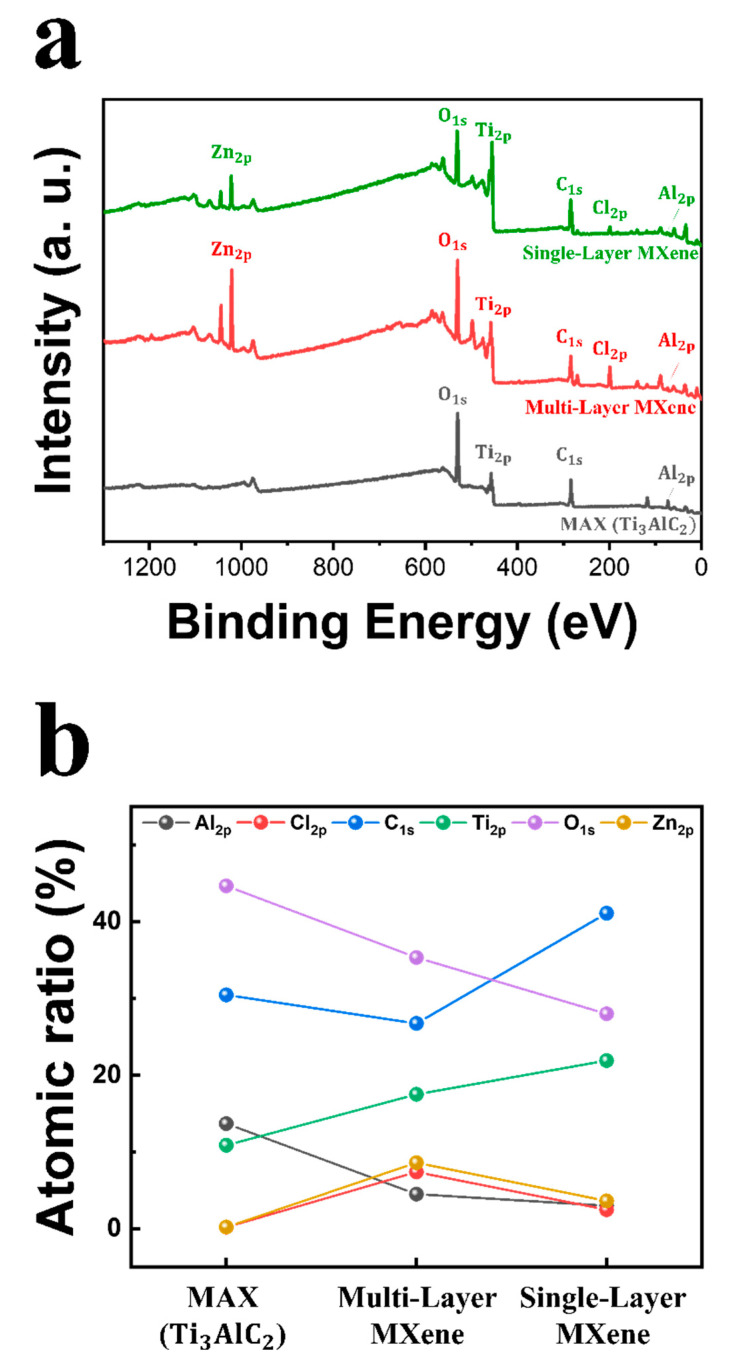
(**a**) XPS survey spectra of MAX (gray), multi-layer MXene (red), and single-layer MXene (green). (**b**) Atomic ratio of XPS survey spectra.

**Figure 13:**
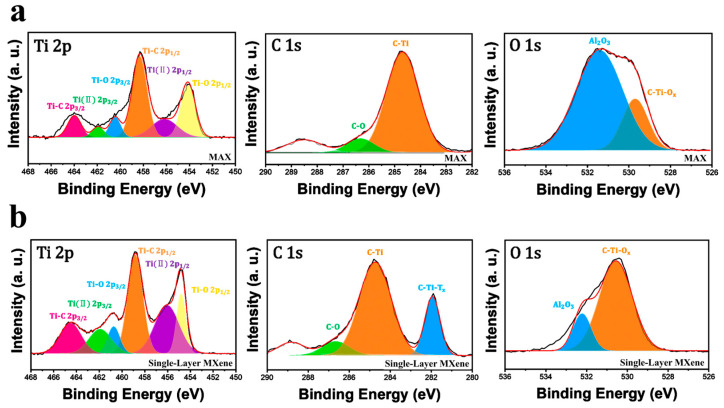
Component peak fitting of XPS spectra of (**a**) MAX phase and (**b**) single-layer MXene.

In practice, the MXene synthesis process results in a random distribution of surface functional groups that are heterogeneously dispersed across the entire sheet. The literature has demonstrated that preparing MXenes with uniform surface terminations remains a significant challenge [63,64]. XPS can provide helpful information on the elemental composition of MXenes. Figure 12 shows the XPS survey spectra and atomic ratio of the samples, and individual spectra are shown in Figure 13. The binding energies of six elements, Al 2p (74.12 eV), Cl 2p (199.01 eV), C 1s (284.8 eV), Ti 2p (58.79 eV), O 1s (530.57 eV), and Zn 2p (1022.21 eV), are displayed in Figure 12a. A detailed comparison of atomic contents after etching demonstrated that the Al content in the multi-layer MXene decreased by 68%, from 13.68% to 4.49%. Additionally, Cl and Zn elements were detected at 7.38% and 8.59%, respectively. In the single-layer MXene, the Al intensity decreased to 2.98% after delamination, indicating the almost complete removal of Al. Cl and Zn were also nearly removed, as shown in Figure 12b. Thus, both the etching and delamination processes effectively removed Al and introduced the Cl and non-fluorinated surface terminations. The narrow and deconvoluted peaks are presented in Figure 13, where the results in (a) correspond to the MAX phase and those in (b) represent the single-layer MXene. In the Ti 2p spectra, the overall peak positions and intensities remain comparable. However, the slight increase in the intensities of the Ti–O 2p_3/2_ and Ti–O 2p_1/2_ peaks indicates a minor degree of oxidation in the MXene. The C 1s spectra reveal the retention of C–Ti bonds following MXene formation, demonstrating the preservation of the titanium carbide framework and the formation of terminal functional groups. Lastly, the O 1s spectra exhibit a pronounced reduction in the intensity of the Al_2_O_3_ peak, confirming the efficient removal of Al and its oxide byproducts.

**Figure 14:**
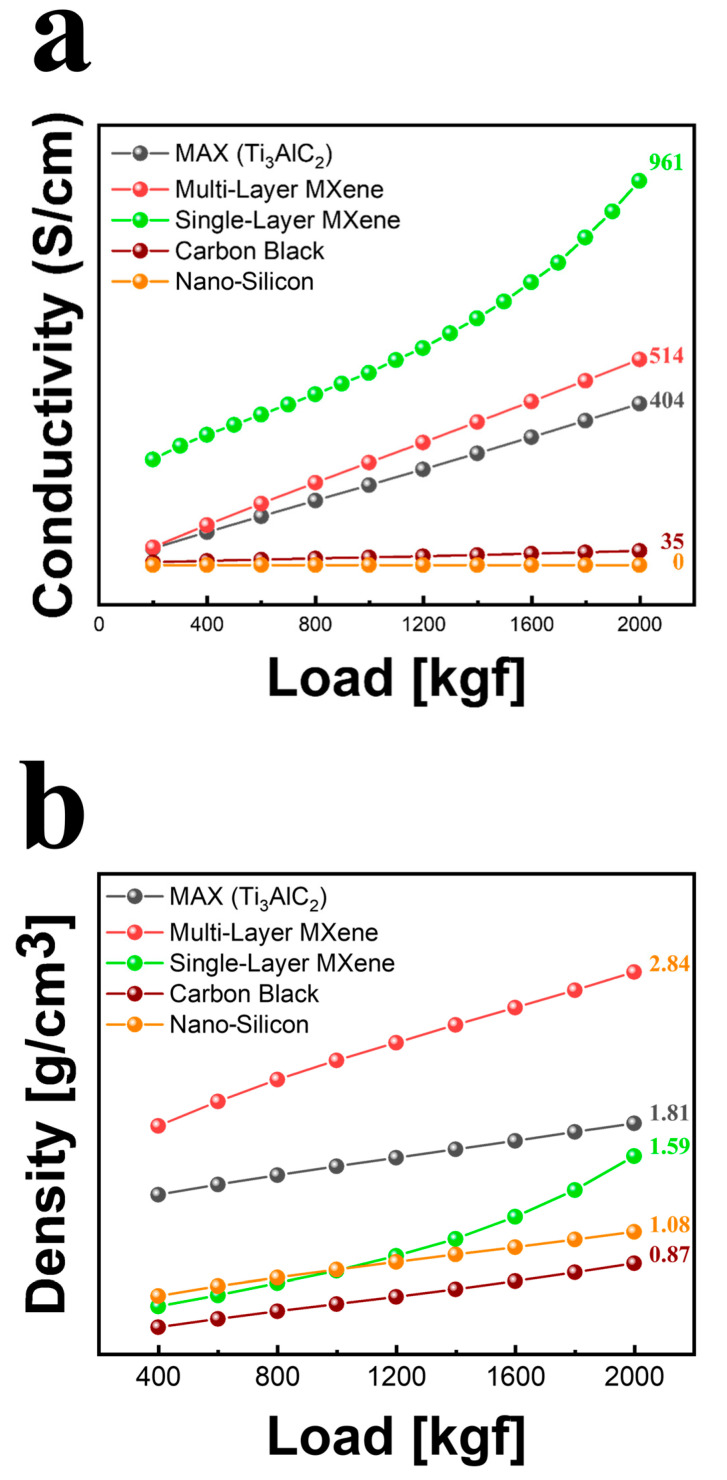
Powder resistivity measures of (**a**) conductivity and (**b**) density under load.

The surface resistance, bulk resistivity, electrical conductivity, and density of powdered MXene and other nanomaterials were measured under unit pressure. Nano-silicon, intended for use as a battery active material, is classified as a semiconductor with a low electrical conductivity of $10^{-4}$ S/cm [65]. Since the powder resistivity measuring system could not measure values below $10^{-3}$ S/cm, the electrical conductivity of nano-silicon was detected as zero (Figure 14). In terms of current, carbon black, the most commonly used conductive additive, exhibited an electrical conductivity of 35 S/cm. In contrast, the electrical conductivity of MAX, the precursor of MXene, was 404 S/cm. After etching, the electrical conductivity of multi-layer MXene slightly increased to 514 S/cm. Single-layer MXene demonstrated the highest electrical conductivity of 961 S/cm. As shown in Figure 14a, it is expected to outperform carbon black as a conductive additive when used in conjunction with nano-silicon and other active materials.

Regarding the density comparisons shown in Figure 14b, nano-silicon and carbon black exhibit similar densities of 0.87 g/cm^3^ and 1.08 g/cm^3^. MAX had a density of 1.81 g/cm^3^, which increased to 2.84 g/cm^3^ after etching to form multi-layer MXene. However, the density decreased to 1.59 g/cm^3^ for single-layer MXene. This suggests that the increased density of multi-layer MXene resulted from residual Al, byproducts, or ions such as Zn or Cl. After the delamination process, where these residues were removed, the density of single-layer MXene decreased accordingly.

### 3.2. Preparation and Battery Applications of Si-MXene/PDA Composite

#### 3.2.1. Preparation of Si-MXene/PDA Composite

**Figure 15:**
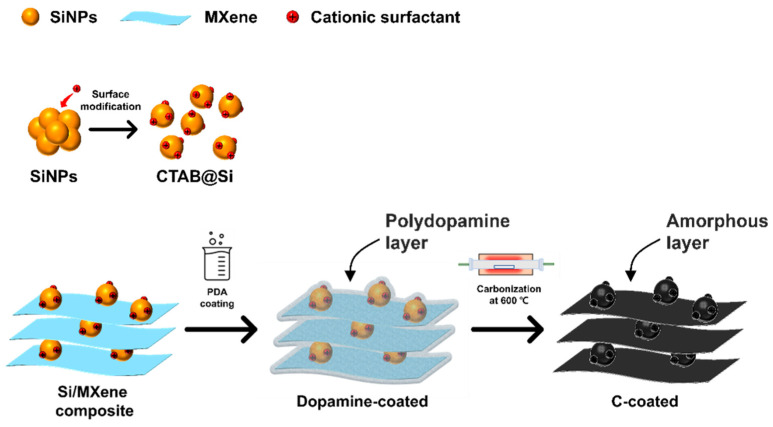
Schematic of the synthesis route of Si-MXene/PDA composite.

**Figure 16:**
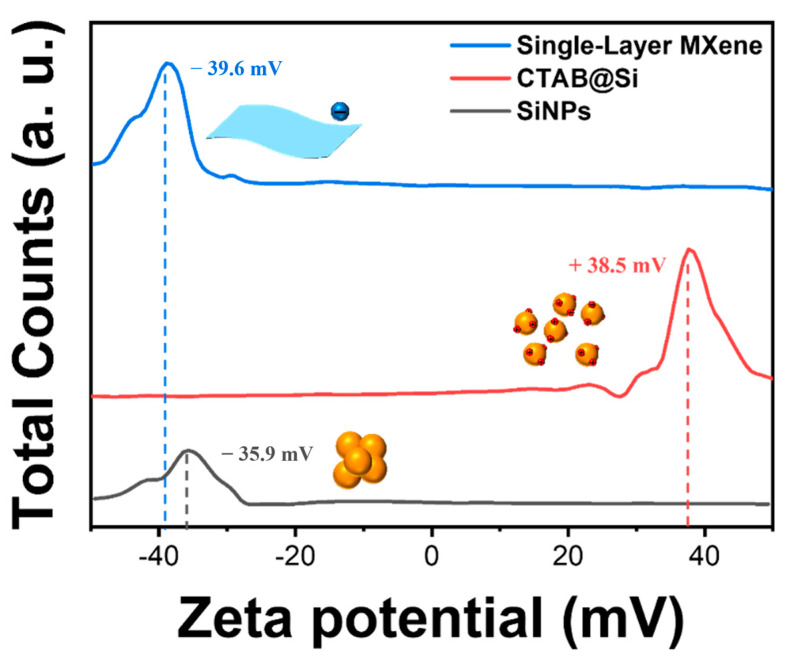
Zeta potential of SiNPs, CTAB@Si, and single-layer MXene.

The synthesis route of the Si-MXene/PDA composite is illustrated in Figure 15. SiNPs with a zeta potential of −35.9 mV were initially subjected to surface modification using CTAB before being combined with MXene. Both the silicon oxide layer of the SiNPs and MXene possess negative charges. CTAB acts as a charge bridge between the silicon oxide layer and MXene, as illustrated in Figure 16. The positively charged silicon prepared through this process was labeled CTAB@Si, with a zeta potential of +38.5 mV. It was combined with MXene, which has a zeta potential of −39.6 mV, via electrostatic interactions. The resultant sample corresponds to the Si-MXene composite, which was subsequently coated uniformly via an in situ polydopamine synthesis. After the final heat treatment, the Si-MXene/PDA composite was successfully prepared.

**Figure 17:**
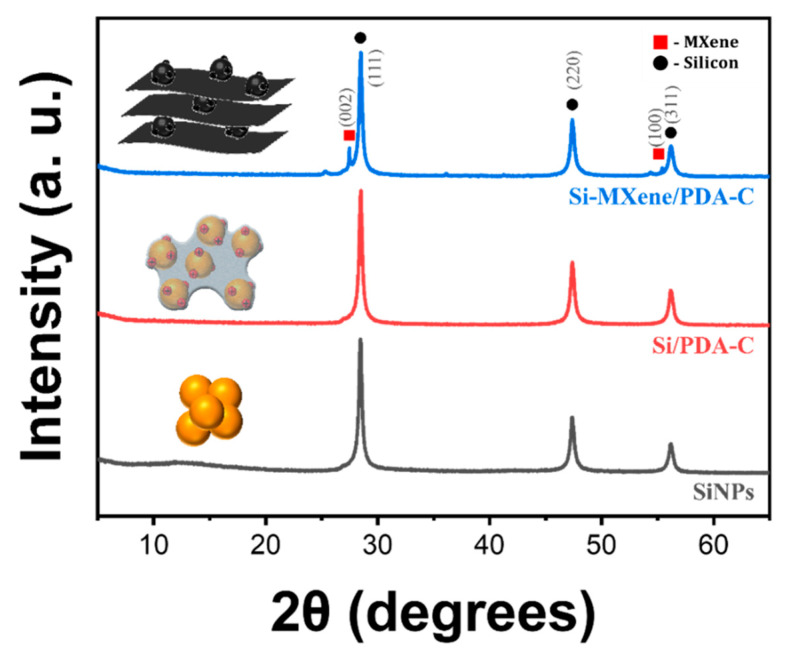
X-ray diffraction patterns of SiNPs (black), Si/PDA (red), and Si-MXene/PDA composite (blue).

The XRD patterns of the SiNP, Si/PDA, and Si-MXene/PDA samples exhibited characteristic diffraction peaks corresponding to the (111), (220), and (311) crystallographic planes of silicon [63]. Si/PDA refers to a sample prepared by creating an aqueous solution of SiNPs in a Tris buffer solution (0.01 M, pH 8.7), followed by the addition of dopamine hydrochloride to induce the polymerization of polydopamine on the surface of SiNPs. These results confirm that the crystalline structure of SiNPs was preserved even after CTAB-assisted surface modification, PDA coating, and thermal annealing. Moreover, no diffraction peaks corresponding to SiC or SiO_2_ phases were observed, indicating that no oxidation-derived byproducts were formed during the synthesis process. For the Si-MXene/PDA composite sample, MXene was identified through its (002) and (100) crystallographic planes at 26.2° and 56°, respectively, demonstrating the successful integration and strong interaction between silicon and MXene shown in Figure 17 [66,67].

#### 3.2.2. Morphology Analysis of Si-MXene/PDA Composite

**Figure 18:**
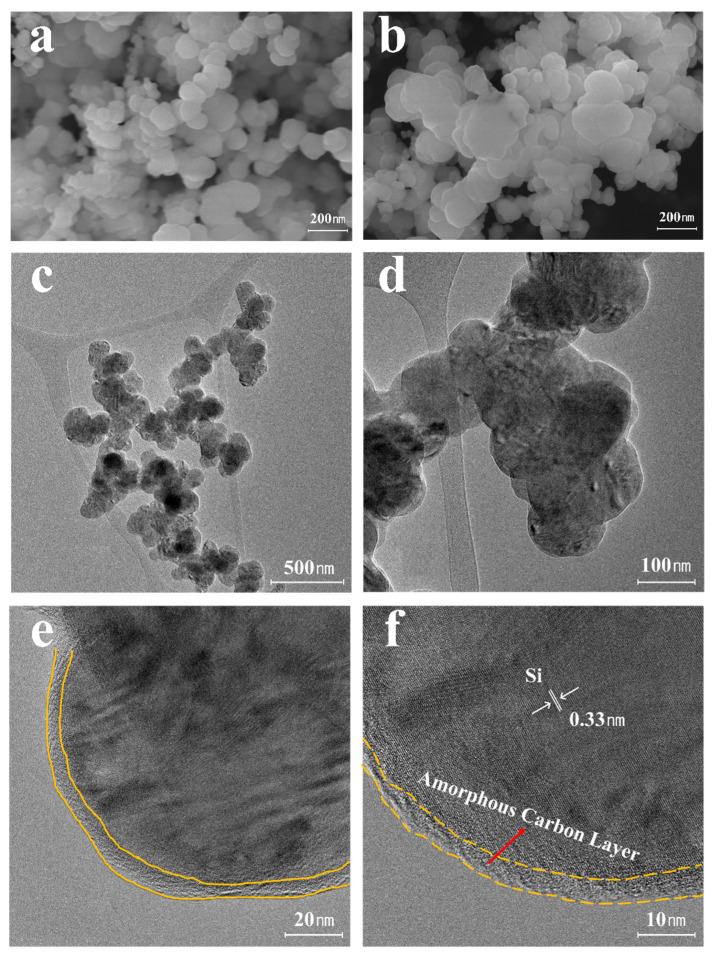
FE-SEM images of (**a**) SiNPs and (**b**) Si/PDA composite. Cs-TEM images of (**c**–**f**) Si/PDA composite.

The morphology of the SiNP and Si/PDA samples were observed using FE-SEM. As shown in Figure 18a, SiNPs with an average diameter of approximately 50.0 nm are detected to form agglomerates. After coating, Figure 18b reveals an increase in particle size due to the deposition of a PDA layer on the surface of the SiNPs. PDA-coated silicon particles typically maintain their original spherical morphology. The images also demonstrate the prevention of particle agglomeration due to the PDA coating layer, which facilitates improved dispersion in electrode matrices. To further investigate the microstructure of the composites, Cs-TEM and EDS mapping analyses were performed. Figure 18c–f confirm the uniform deposition of the PDA layer on the SiNPs, with each nanoparticle encapsulated by an amorphous carbon layer of approximately 5–10 nm thick. The lattice spacing of 0.33 nm observed in the Cs-TEM images corresponds to the (111) crystal plane of silicon [68].

**Figure 19:**
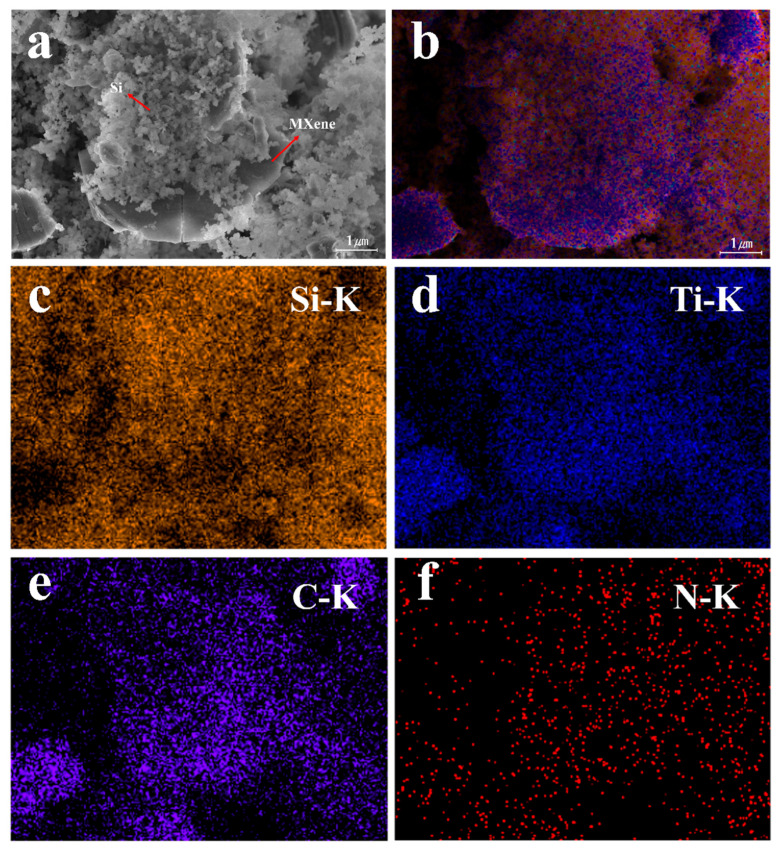
FE-SEM image of (**a**) Si-MXene/PDA and (**b**–**f**) EDS mapping images.

Figure 19 illustrates the hierarchical nanostructure of the Si-MXene/PDA composite. It can be observed that SiNPs are well attached to the surface of the multi-micron MXene sheets. MXene can enhance the electrical conductivity of nano-silicon and improve interfacial adhesion, mitigating side reactions between the active material and the electrolyte and facilitating the formation of a stable solid electrolyte interphase (SEI) layer. The PDA coating layer serves as a protective barrier, further enhancing the structural stability of the anode material [69]. EDS mapping reveals a homogeneous distribution of C and N elements within the Si-MXene/PDA composite, demonstrating the robust integration of SiNPs with MXene and the successful application of the PDA coating (Figure 19b–f).

The thickness of the PDA layer is crucial, as a uniform coating effectively buffers the volume expansion of silicon nanoparticles, preventing mechanical degradation during charge–discharge cycles. While thicker PDA layers offer enhanced mechanical support, they may increase ionic diffusion resistance. Therefore, an optimal thickness of 5–10 nm, as observed in our study, achieved a balance between stability and conductivity. The carbonization process transforms the PDA layer into a nitrogen-doped carbon (N-doped carbon) shell. Under controlled carbonization conditions of 600 °C for 5 h, the PDA layer developed into an amorphous carbon structure that enhanced electrical conductivity while maintaining flexibility to accommodate volume changes. As will be explained in the following section on the electrochemical performance of Si-MXene composite anode, the carbon shell not only reduced side reactions with the electrolyte, thereby forming a stable solid electrolyte interphase (SEI), but also compensated for the inherently low conductivity of SiNPs, ensuring efficient charge transfer.

### 3.3. Electrochemical Performance of Si-MXene Composite Anode

**Figure 20:**
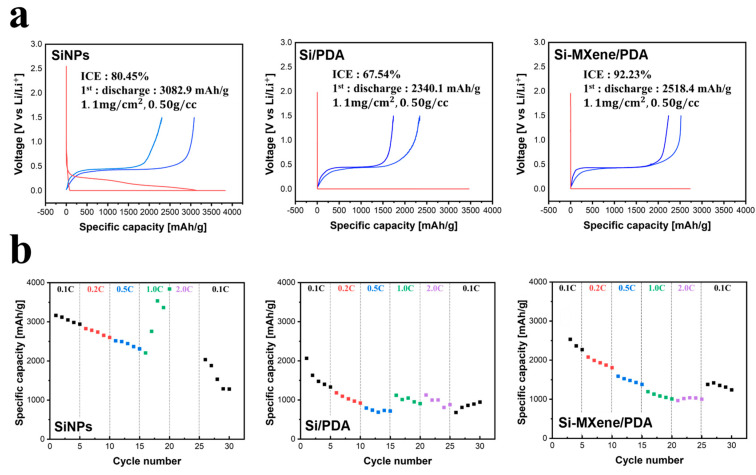
(**a**) Charge–discharge curves and (**b**) current rate graphs of lithium-ion batteries with SiNP, Si/PDA, and Si-MXene/PDA composite anodes at a current density of 0.1 A/g.

Figure 20a illustrates the capacity and initial Coulombic efficiency (ICE) of the lithium-ion batteries (LIBs) with SiNP, Si/PDA, and Si-MXene/PDA composite electrodes at a current density of 0.1 A/g. ICE corresponds to the ratio of the initial discharge capacity to the initial charge capacity. All three anodes were fabricated with a loading level of 1.1 mg/cm^2^ and an electrode density of 0.50 g/cc, which was followed by performance analysis to achieve uniform densities across all electrodes, based on which the thickness was adjusted. The LIB with an SiNP anode exhibited an initial discharge capacity of 3082.9 mAh/g, which is appropriate considering the theoretical capacity of silicon and the 80% silicon content relative to the total anode mass [70,71]. The LIB with an Si/PDA anode, coated with polydopamine and carbonized but without MXene, displayed a lower initial discharge capacity of 2340.1 mAh/g. In contrast, the LIB with an Si-MXene/PDA anode demonstrated an initial discharge capacity of 2518.4 mAh/g, higher than that of the Si/PDA anode. The ICE of the SiNP anode was 80.45%, which aligns with typical values for silicon anodes. The Si/PDA anode showed a significant reduction in ICE to 67.54%, whereas the MXene-containing Si-MXene/PDA anode exhibited a much higher ICE of 92.23%, comparable to that of conventional graphite anodes [72]. These results indicate that the polydopamine coating and subsequent carbonization, which form a carbon layer on the nano-silicon surface, negatively affected the initial charge–discharge efficiency. However, combining nano-silicon with MXene, followed by polydopamine coating and carbonization, significantly improved the initial charge–discharge efficiency. This improvement was attributed to the high electrical conductivity of MXene.

Figure 20b represents the C-rate performance graphs for each LIB sample, featuring electrodes made with SiNP, Si/PDA, and Si-MXene/PDA composites. The tests were conducted by gradually increasing the current rate from 0.1 C to 2 C at five-cycle intervals. At 2 C, the charge-and-discharge time was set to 30 min, enabling the evaluation of the stability of each anode under high-speed charging conditions [73,74]. The LIB cells with SiNP and Si/PDA anodes displayed instability even at 1 C, which corresponds to a 60 min charge/discharge time. In contrast, the cells featuring the Si-MXene/PDA composite anode showed some reduction in capacity retention at 1 C and 2 C, yet they maintained stable performance, achieving a reversibility of over 65% at 0.1 C. This indicates that anodes composed solely of SiNPs were highly unstable under high current rates, as evidenced by the breakdown observed in the C-rate performance. The Si/PDA anode, prepared through polydopamine coating and carbonization, demonstrated potential for enhancing the stability of nano-silicon anodes [75]. Despite resulting in a lower ICE, it showed a noticeable improvement in overall structural stability. Finally, the Si-MXene/PDA composite sample, leveraging the high electrical conductivity of MXene to offset the low conductivity of nano-silicon, exhibited both a higher ICE and superior stability under high current rates. While further optimization may be necessary, the Si-MXene/PDA composite sample exhibited the most desirable characteristics for advanced anode applications [76].

**Figure 21:**
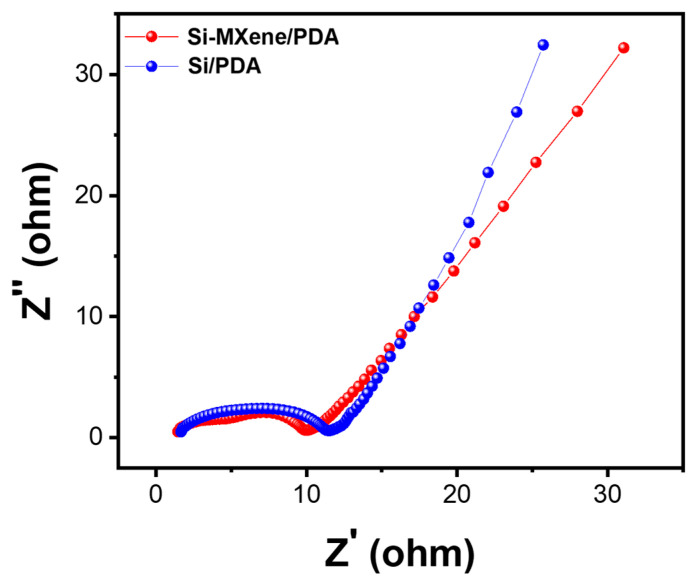
Electrochemical impedance spectroscopy analysis of Si/PDA and Si-MXene/PDA electrodes.

Figure 21 illustrates the results of EIS analysis, confirming the reduction in internal resistance and the improvement in charge transfer kinetics of the Si-MXene/PDA composite electrode. The Nyquist plot reveals that the Si-MXene/PDA electrode exhibited a smaller semicircle in the high-to-medium frequency region compared to the Si/PDA electrode, indicating a lower charge transfer resistance. This improvement is attributed to the enhanced electrical conductivity provided by the MXene component. Furthermore, in the low-frequency region, the Si-MXene/PDA electrode demonstrated a steeper slope, suggesting improved lithium-ion diffusion kinetics. This enhancement likely resulted from the increased interlayer spacing and superior ion transport properties of MXene, facilitating more-efficient lithium-ion mobility. The combination of these factors effectively addressed the inherent challenges of silicon-based anodes, which typically suffer from poor electrical conductivity and sluggish ion diffusion. These findings highlight the crucial role of MXene in mitigating the limitations of silicon anodes and enhancing their electrochemical performance [77]. The observed reduction in charge transfer resistance and improved lithium-ion transport confirm that the Si-MXene/PDA composite offers significant advantages for lithium-ion battery applications.

**Figure 22:**
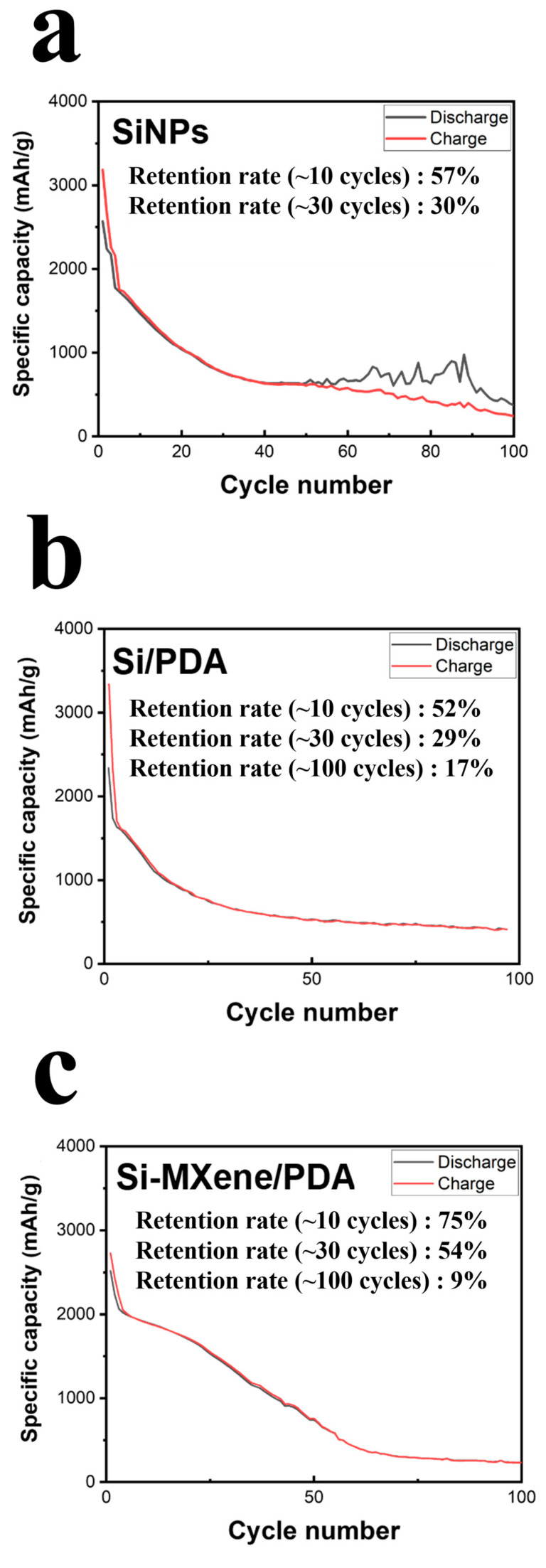
Cycling performance of lithium-ion batteries with (**a**) SiNP, (**b**) Si/PDA, and (**c**) Si-MXene/PDA composite anodes.

Figure 22 compares the cycling performance of each LIB sample, including anodes made from SiNPs, Si/PDA, and the Si-MXene/PDA composite. The SiNP anode cell exhibited a high initial discharge capacity, exceeding 2500 mAh/g. However, it showed the most rapid capacity fading, with retention rates of 57% after 10 cycles and 30% after 30 cycles. After 50 cycles, the charge–discharge curves indicated a loss of reversibility. This result suggests that using nano-silicon alone as an anode material lead to significant issues, such as volume expansion and weak adhesion, which likely resulted in delamination from the copper current collector [78,79]. The Si/PDA anode displayed a lower initial capacity compared to that of the SiNP anode. However, it demonstrated reversible charge–discharge behavior despite capacity fading over up to 100 cycles. The retention rates after 10, 30, and 100 cycles were 52%, 29%, and 17%, respectively. Although the performance after up to 30 cycles was not significantly different from that of the SiNP anode, the Si/PDA anode maintained mechanical integrity beyond 50 cycles, indicating improved structural stability. The Si-MXene/PDA composite anode also exhibited a lower initial capacity compared to the SiNP anode but achieved superior retention rates due to its higher initial ICE. The retention rates after 10, 30, and 100 cycles were 75%, 54%, and 9%, respectively. This result highlights MXene’s ability to compensate for the low electrical conductivity of nano-silicon by providing a conductive network. Furthermore, the Si-MXene/PDA composite anode demonstrated the most stable slope among the three samples, suggesting promising potential for further optimization.

**Figure 23:**
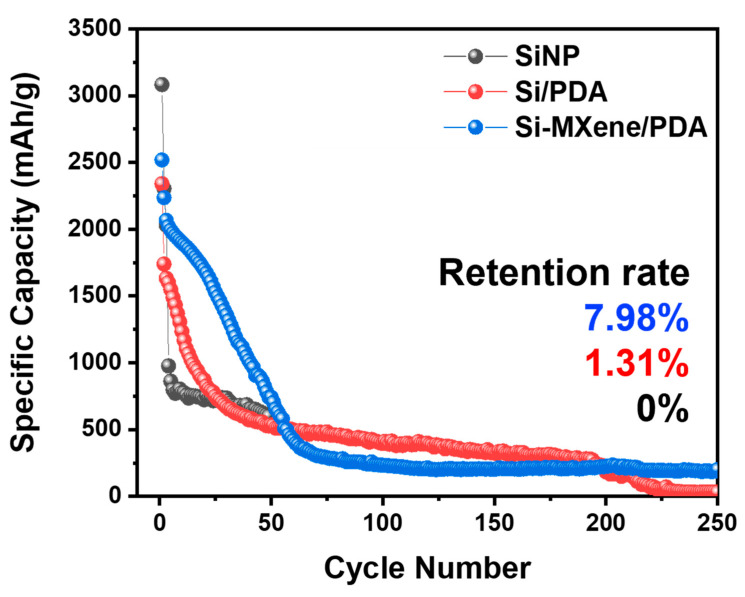
Long cycling performance of lithium-ion batteries with SiNP, Si/PDA, and Si-MXene/PDA composite anodes at a current density of 0.1 A/g.

Figure 23 presents the long-term cycling stability of the three anodes, evaluated over 250 cycles. The SiNP anode showed the most rapid capacity decay, failing to sustain stable cycling beyond 50 cycles. The Si/PDA anode underwent rapid capacity fading during the initial cycles. However, it maintained a stable slope between 50 and 200 cycles, with a retention rate of 1.31% after 250 cycles. The Si-MXene/PDA anode outperformed the Si/PDA anode in both capacity and slope stability. It maintained a highly stable slope during the initial cycles and achieved a retention rate of 7.98% after 250 cycles. Although the final capacity approached the level of graphite-based anodes (~300–400 mAh/g), the Si-MXene/PDA anode demonstrated superior retention compared to the other anodes. These findings suggest that combining nano-silicon with highly conductive MXene, along with applying PDA coating and carbonization to form a carbon nano-layer, significantly enhanced ICE and cycling stability [80].

## 4. Conclusions

In this study, MXene was successfully synthesized using an eco-friendly molten salt method, addressing the environmental and safety concerns associated with conventional HF-based synthesis. The eco-friendly MXene was then utilized in conjunction with nano-silicon to develop advanced composite anodes for energy storage applications. By integrating MXene with nano-silicon, followed by the application of polydopamine coating and carbonization, the resulting Si-MXene/PDA composite demonstrated enhanced electrochemical performance and stability. The Si-MXene/PDA anode exhibited superior ICE and excellent stability under high current rates, compared to SiNP and Si/PDA anodes. This improvement can be attributed to MXene’s high electrical conductivity, which effectively compensates for the low conductivity of nano-silicon, as well as the structural reinforcement provided by the polydopamine coating and carbonization process. These synergistic effects ensured stable charge–discharge performance even at high C-rates, highlighting the potential of this composite anode for advanced battery applications. While the results demonstrate the promise of eco-friendly MXene synthesis and its integration into composite anodes, further optimization is required to improve scalability and enhance long-term cycling stability. Nevertheless, this study provides a strong foundation for the development of sustainable, high-performance anode materials, paving the way for environmentally conscious advancements in energy storage technologies.

## Data Availability

Data are contained within the article.
